# Revisiting metallization boundary of warm dense helium in a wide *ρ*-*T* regime from ab initio study

**DOI:** 10.1038/srep41885

**Published:** 2017-02-03

**Authors:** Wei Zhang, Zhiguo Li, Zhijian Fu, Jiayu Dai, Qifeng Chen, Lingcang Cai

**Affiliations:** 1 Laboratory of Shock Wave and Detonation Physics, Institute of Fluid Physics, Chinese Academy of Engineering Physics, P.O. Box 919-102, Mianyang 621900, Sichuan, P. R. China; 2 School of Science, Southwest University of Science and Technology, Mianyang, 610064, Sichuan, P. R. China; 3 College of Science, National University of Defense Technology, Changsha, 410073, Hunan, P. R. China

## Abstract

The knowledge of the metallization of warm dense helium has important implications for understanding the thermal histories, stellar structure and magnetic field environment of giant planets. However, it is also a pendent scientific topic. For a revisiting into the properties of warm dense helium, we performed extensive quantum Langevin molecular dynamic simulations and electronic structure calculations to study helium over a very wide range of density (*ρ* = 1~24 g/cm^3^) and temperature (*T* = 10~160 kK). The dependencies of helium band gap on *ρ* and *T* were presented and a metallization boundary of helium was thus determined by gap closure. Such a boundary is further identified by the calculated electrical conductivity and optical reflectivity based on Kubo-Greenwood formula: along the boundary, the electrical conductivities are found to be 7.0 × 10^5^~1.3 × 10^6^ Ω^−1^ m^−1^ and the optical reflectivity value at 532 nm is about 0.55, which are typical values for true metal.

As the second most-abundant chemical element in the universe, helium makes up a large fraction of giant gaseous planets^1^ and most extrasolar planets^2^ which have been discovered so far. All of these giant planets are likely to be in a fluid state throughout. The investigation of the phase diagram of fluid helium in warm dense condition is not only of fundamental interest but also an indispensable prerequisite to model these astrophysical objects. Of particular interest is the domain on the phase diagram where helium makes the transition from an insulator to an electrical conductor. Because the metalized density-temperature profile of helium are relevant for modeling the opacity and the fastest cooling rate of He-rich white dwarfs (WDs) which are influenced by the treatment of the ionization of He in the warm dense regime^3^,^4^. Besides, the electrical conductivities of H and He are important for understanding the generation of magnetic fields in the deep interiors of giant planets in the solar system and in the about 100 extrasolar planets discovered to date^5^.

However, unlike other low Z molecular fluids such as H_2_(D_2_), O_2_, and N_2_^6^,^7^, helium has not yet been achieved its metallic state experimentally. For cold solid helium, earlier diffusion quantum Monte Carlo (DMC) calculations predicted that the band gap closed at a density of 21.3 g/cm^3^ and a pressure of 25.7 terapascals^8^. By considering the electron-phonon coupling effects, a recent theoretical results by Monserrat *et al*. gave a much higher metalized pressure of 32.9 TPa^9^. Such a high pressure is far from experimental reach within current static pressure technique. However, in shock wave experiments, some typical low Z molecular fluids achieved their metallic states under pressures only about 100 GPa^6^,^7^ indicated that metalized pressure could be significantly reduced by the high temperature produced in shock wave compression^6^. Thus, some possible metallization of dense fluid helium have also been studied by shock wave experiments. The measured electrical conductivity of dense fluid He under multiple shock compression could be achieved to typical liquid alkali metals and the metalized density was estimated to be around 1 g/cm^3^ 10,11 which was identified by quantum molecular dynamics simulation of the day^12^. Combining diamond-anvil-cell and laser-driven shock wave techniques, a recent experimental work by Celliers *et al*. reported that the hot dense He could become metallic above about 1.9 g/cm^3^, this conclusion was obtained by fitting their optical measurements of reflectivity with a simple semiconducting Drude model without considering the thermal effects^13^. The reported two value of the metalized density of helium are surprisingly smaller than the prediction by the Goldhammer-Herzfeld (GH) criterion (7.7 g/cm^3^) or by the Mott criterion (4.7 g/cm^3^)^13^. Another latest experiments have been performed on the noble gases Xe, Ar, Ne, and He in the laser-heated diamond anvil cell to observe their insulator-to-conductor transformations, time domain spectroscopy of thermal emission was employed to determine temperature and establish corresponding sample optical properties^14^. The results indicate that helium takes a very wide band gap about 11 eV at a density of about 1.5 g/cm^3^ and temperature about 11 kK. At this thermodynamical conditions, helium is still far from being metallized which is very different from the results by Celliers *et al*.^13^. By taking into account a temperature dependence for the gap energy derived from the existing ab initio calculations^15^, Soubiran *et al*. reconsidered Celliers *et al*.’s experimental data and predicted a much higher metalized density at about 10 g/cm^3^ under a temperature about 3 eV^16^. Actually, previous theoretical study had also shown that the metallization pressure of dense fluid helium presented a strong temperature dependence^17^.

Although some theoretical and experimental results have been reported on the metallization of fluid helium, a reliable metallization boundary covered a wide *ρ* − *T* domain in the phase diagram of helium is still missing. In this work, by performing extensive quantum Langevin molecular dynamics (QLMD) simulations and static electronic calculations, we have built this metallization boundary of warm dense helium. In addition, wide range Ddirect-Current (DC) conductivity and optical reflectivity data were obtained which are essential input for modeling He-rich astrophysical objects.

## Methods

### Quantum Langevin molecular dynamics

In ordinary QMD, ions are moved by electronic force according to Newton equation for every dynamic step. However, in the warm and hot dense matter regime, the behavior of ions dynamic likes classical Brownian motion due to frequent electron collisions. In order to resolve this issue, our QLMD simulations introduced the electron-ion collisions induced friction in the Langevin equation for ionic dynamics i.e. 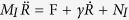, where *F* is the force obtained from density functional theory calculation, *R* is the position of ions, *γ* is a Langevin friction coefficient, *N*_*I*_ is a Gaussian random noise corresponding to *γ*. Such a treatment for ions dynamic was implemented in modified Quantum ESPRESSO package^18^. Within the framework, we have obtained the equation of states(EOS) and the transition of electronic structures of the materials from condensed matter to ideal plasma gas regime^19^, and some novel structures of iron characterized by the ionic clusters with electron bubbles were found^20^. As the errors for forces with large convergence tolerance can be taken as the noises of Langevin dynamics, the efficiency of Born-Oppenheimer molecular-dynamics simulations can thus be improved significantly^21^. Therefore, it is possible to perform a molecular dynamics within dense temperature-density points sampling.

In our QLMD simulations, the cubic cells contained 125 helium atoms, and the NVT ensemble was employed, the simulations were performed with 6000 time steps. The electron wave functions were calculated using norm-conserving pseudopotential with a cutoff energy of 200 Ry and the generalized gradient approximation(GGA) parameterizated by PBE^22^ to calculate exchange and correlation energy. For the density of helium above 12 g/cm^3^, a full Coulomb potential was employed. Because we found the ionic dynamic and structure information given by norm-conserving pseudopotential presents non-physics-based variation: the self diffusion coefficient become larger and larger as the density enhanced which obviously lost the physical truth. At the higher density, the results from full Coulomb potential can give a correct tendency. In the Brillouin zone during the QLMD simulation, only the Γ-point was sampled. All these parameters were tested carefully which could ensure a well convergence of pressure, internal energy and especially the radial pair distribution function *g(r*).

### Dynamic conductivity

The electrical and heat currents characterize the linear response of warm dense helium to external electrical field and temperature gradient, the key to calculate electronic transport properties is the kinetic coefficients based on the Kubo-Greenwood formula^23^,^24^,


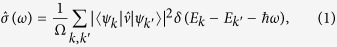


where Ω is the volume of the unit cell, *E*_*k*_ are the electronic eigenvalues, and 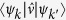 are the velocity matrix elements. In the Chester-Thellung version^25^, the kinetic coefficients *L*_*i,j*_ can be evaluated by,


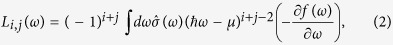


where *f(ω*) is the Fermi-Dirac distribution function and *μ* is the chemical potential. Then the frequency dependent electronic thermal conductivity *K* and electrical conductivity read,


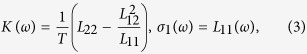


Those formulation are implemented in the ABINIT code^26^, and have lead to good results for liquid aluminum^27^ and hot dense hydrogen^28^. The consistency of the electrical conductivity is checked via the sum rule^23^,


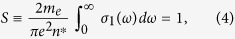


where *m*_*e*_ is the electron mass and n* is the electronic density. We maintained a sufficient number of bands (up to 600) to satisfy the sum rule to at least within 5%. For selected statistically independent atomic configurations, self-consistent ground-state calculations were performed to get the detailed electronic structure including electronic density and the Fermi-Dirac occupations. Then non self-consistent field (SCF) calculation was performed from the previous electronic density and wave functions which gave the needed Kohn-Sham eigenvalues. From the wave functions of the previous non-SCF calculation, the derivative of the Hamiltonian with respect to the wave vector for the three directions could be derived from the calculations of response function. The electronic calculations were performed in the GGA with the exchange-correlation energy functional of the Perdew, Burke, and Ernzerhof (PBE)^22^. The norm-conserving pseudopotential used in this case was generated by the Troullier-Martins method^29^. Orbitals were expanded in plane waves with a cutoff energy of 200 eV. A 3 × 3 × 3 Monkhorst-Pack *k*-points mesh was used.The total energy convergence had been checked against the plane-wave cut off energy and number of *k*-points to obtain a convergence up to 0.1 meV. The tolerance on wavefunction squared residual was chosen to be 10^−22^ in non-SCF calculation. To get statistically converged results, at given *ρ* − *T* point, the electrical conductivity is averaged over five snapshots picked up in every 200 MD steps from the thermalized part of the simulations. The time scale of two adjacent snapshot is 50 fs which is much longer than the correlation time of helium during the MD simulation, so it can ensure a statistically independent sampling.

### Optical properties

The imaginary part of conductivity *σ*_2_(*ω*) can be obtained from the real part of the electric conductivity via the Kramers-Kronig relation as,


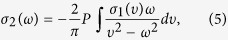


where *P* is the principal value of the integral and *υ* is the frequency. The complex dielectric function *ε(ω*), index of refraction *n(ω*), coefficient of extinction *k(ω*), and reflectivity *R(ω*) can be obtained from the following equations,


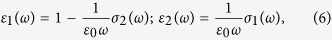



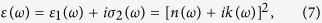



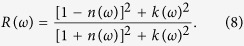


## Results and Disicussion

### Helium gap in the warm dense matter regime

**Figure 1:**
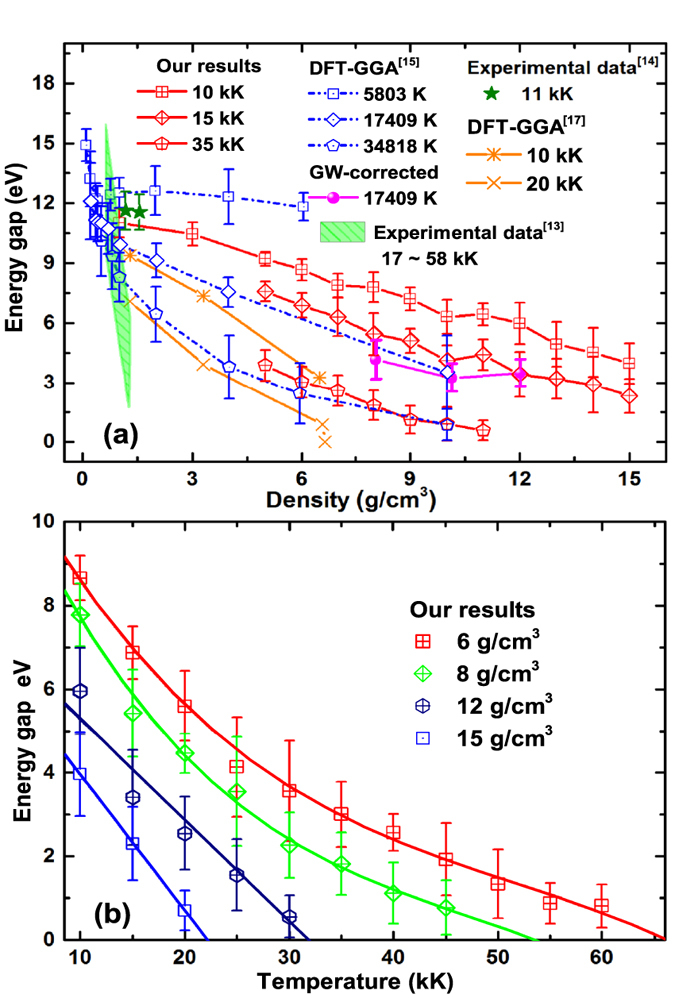
The evolution of band gap with density (**a**) and temperature (**b**) with other experimental data and theoretical results for a comparison.

Helium crystal in the ground state is known to be an electrical insulator with a rather large gap about 20 eV between the highest occupied and the lowest unoccupied electron bands. Compression and calefaction can shrink the gap to zero gradually, i.e. the helium is transformed to be metallic. In the present electronic structure calculations after QLMD simulations, denser *k*-point mesh of 8 × 8 × 8 were employed to ensure the convergence of the electronic energy gap values. Figure 1(a) shows the behavior of band gap with densities under three different temperatures calculated by density functional theory (DFT) within GGA scheme, comparing with other theoretical and experimental results. All the gaps were obtained by averaging at least ten snapshots along the whole trajectory and the error bars were the mean square deviation of them. It can be found that our results agree well with those from Kowalski *et al*.^15^. However, the calculated gaps by Stixrude *et al*.^17^ show much stronger dependence on the densities, which yields a much lower metallization density at finite temperature. Without a consideration about temperature effects, Celliers *et al*. suggested the lowest density condition for helium metallization at 1.9 g/cm^3^ 13. The dependencies of gap on temperature are illustrated in Fig. 1(b), it can be found that the gap shrinks quickly when the temperature is below about 30 kK and then the trend become weak. From the analyses of electronic density of states, we find that closure of the band gaps originates primarily from both the pressure induced broadening of valence band and temperature (actually disorder extents) induced shift of the conduction band to lower energy, which is similar to the findings by former electronic calculations^17^.

As a ground state approach, DFT method is formally not appropriate for handling excited states and underestimates band gaps of semiconductors systematically^30^. This inadequacy of DFT methods is associated with the discontinuity in the exchange-correlation potential when the system adds one electron^31^,^32^ and it occurs even if the exact functional is employed^33^. In the study by Kowalski *et al*.^15^, they further used an exact exchange hybrid PBE0 density functional^34^ as well as the GW approximation to estimate the uncertainties on the electrical and optical properties resulting from the GGA approximation, they found that the GW correction added 6 eV to the GGA band gap while the hybrid functional calculation increased the GGA band gap by about 3 eV. However, both the GW and hybrid calculations were performed at zero electronic temperature and, as such, were upper bounds to the band gap correction. Besides, it is noted that recent theoretical results based on the all-electron quasiparticle self-consistent GW approximation and Keldysh time-loop Green’s function approach show that DFT underestimates the gap by considerably less than previously thought at finite temperature^35^. As shown in Fig. 1(a), the GW results at the high density and temperature are very close to our GGA results and the difference between them become negligible as the density increased further.For checking the validity of our GGA results at finite temperature, we have also performed an electronic band structure calculation with the Heyd-Scuseria-Ernzerhof (HSE) screened hybrid functional^36^ for helium at 10 g/cm^3^ and 35 kK for just one ionic configuration (a point near the metallized boundary), the calculated band structure is very similar to the one given by GGA in general. In the vicinity of the Fermi level, the GGA results show a just closed gap and the HSE band structure gives a very small gap about 0.043 eV. The difference between them is very small at this temperature. Moreover, our GGA results and the recent experimental gap data estimated by McWilliams *et al*. from optical measurement^14^ at about 11 kK are in reasonable agreement with each other. So the metalized thermodynamic conditions under high density and temperature determined by GGA band gap closure within DFT framework in this study can be considered to be highly reliable.

**Figure 2:**
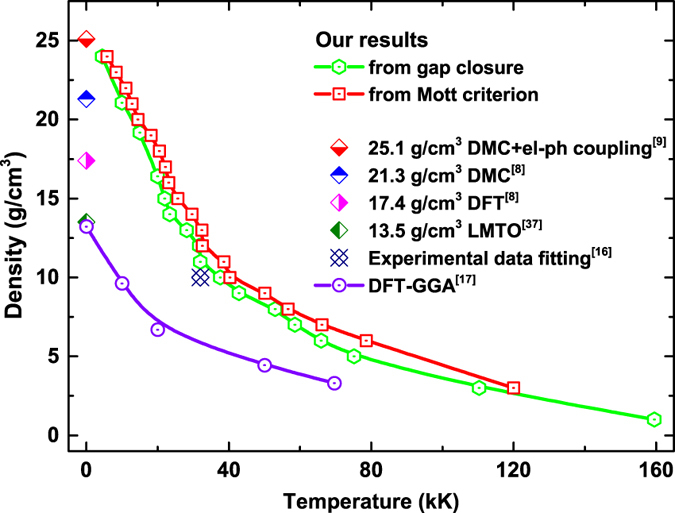
Calculated metallization boundary of fluid helium together with other theoretical predictions for a comparison.

After the hundreds of QLMD simulations for different densities and temperatures, we chose more than 1000 ionic configurations from the dynamic trajectories for electronic structure calculations and finally determined the metalized boundary. The boundary is illustrated in Fig. 2, it can be found that the metalized temperature (more than 10 eV) required for available density of fluid helium in laboratory is much higher than previous theoretical prediction^12^,^17^ and experimental estimation^13^, which indicates temperature activated metallization of low density helium. However, the metalized temperature at density of 10 g/cm^3^ in this work is about 37581 K which is close to 3 eV given by Soubiran *et al*.^16^ who fitted the experimental data from Celliers *et al*.^13^ by introducing the effect of temperature on the gap energy. What’s more interesting is that if we extrapolate the obtained metalized boundary to zero temperature and the corresponding density is would be 26 g/cm^3^, which agrees very well with recent calculation for solid helium by Monserrat *et al*.^9^. In their work, a full consideration of both the electron-phonon coupling effect and thermal expansion using nonperturbative approach was taken. At this stage, it is believed that the previous results for the zero temperature metallization density of solid helium from ordinary DFT^8^ and linear-muffin-tin-orbitals (LMTO) method^37^ are seriously underestimated. All these results suggest a thinner metallic He layer in the interiors and correspondingly larger insulating layer in the outer region of WDs than the previous predictions. Therefore, new considerations should be taken in modeling energy transport in WDs which is essential for understanding of the cooling process and estimating the age of WDs.

Considering the variation characteristic of the gap in wide temperature and density domain, we propose a density and temperature dependent formula for the helium gap value: *E*_*g*_ = *Ae*^−*T/B*^ + *Cρ* + *D*, where *A* = 13.066 eV, *B* = 35611.56 K, *C* = −0.512 eV g^−1^cm^3^ and *D* = 0.793 eV. With such a formula, one can reproduce almost all our calculated gaps and the metalized boundary rather perfectly. Compare with the results given by Soubiran *et al*.^16^, our formula can be established in a much more wider range of density (*ρ* = 1~24 g/cm^3^) and temperature (*T* = 10~160 kK).

### Electrical transport properties

**Figure 3:**
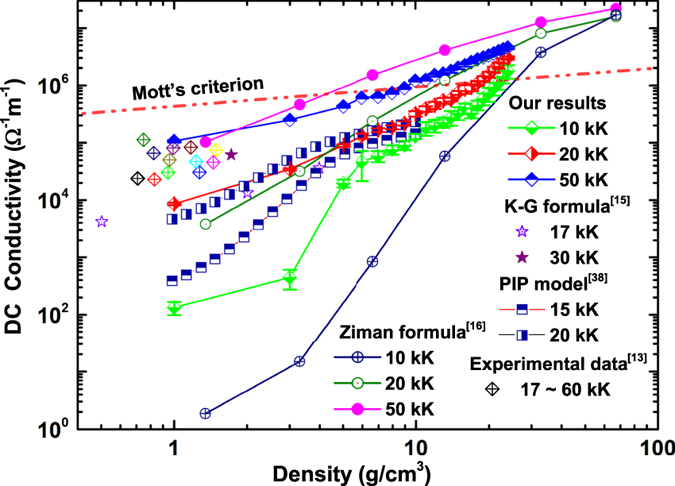
The variation of DC conductivity with density at different temperatures.

For a further understanding of the metallization of warm dense helium, electrical conductivities of helium are usually served as important theoretical supports as well as experimental evidences. The DC electrical conductivity was extracted by extrapolating dynamic conductivity to *ω* = 0. The variation of the DC conductivity along the 10, 20, and 50 kK isotherms are shown in Fig. 3. Comparing with other theoretical results obtained by Ziman formula^13^, Kubo-Greenwood(K-G) formulation^15^, and partially ionized plasma model(PIP) within COMPTRA04 code^38^, one can find a reasonable agreement among them with the exception of the underestimation of DC conductivity given by the Ziman formula at 10 kK. Moreover, our calculated electrical conductivities of helium at 1 g/cm^3^ with temperatures of 20 and 50 kK are also quite consistent to the reported experimental data from Cellier *et al*.^17^ at similar condition^13^. For most cases in the metalized regime, the dependencies of dynamic conductivity on photon energy exhibit a Drude-like form (
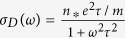
, with n* and *τ* representing the conducting electron number density and relaxing time) which suggests the nearly free-free nature of the system, and some cases show the Drude-smith-like form^39^ (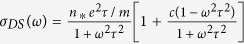, where *c* is a parameter of the memory effect of the successive collision) with the maximum peak being shifted to higher energy. By fitting the frequency dependence of dynamic conductivity to these two forms, one can yield the relaxing times of electrons are about 2~3 × 10^−17^ s and the conducting electron number density about 1~7 × 10^30^ m^−3^ which are typical values for true metal. In addition, the oscillator-strength sum rule on *σ*_1_(*ω*) can give the plasma frequency *ω*_*p*_ (
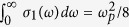
) which can be expressed by 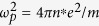, as done in recent work by Saitov *et al*.^40^. In this way, the estimated conducting electron number densities are somewhat smaller but still comparable to those given by the Drude model fitting.

Using these values of n* and *τ*, it is possible to estimate the mean free path *λ* of the conduction electrons. Since the electron inside the material can be degenerate, we can estimate the effective velocity *ν*_*eff*_ of the conduction electrons by the expression 
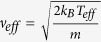
, where 
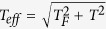
, in this formula, *T*_*F*_ is the Fermi temperature which can be calculated by 
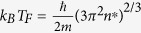
, the mean free path *λ* is given by *λ* = *τν*_*eff*_. We obtained the mean free paths take a order of 10^−11^ m comparable to the average interatomic distance in simulation box which suggests a strong electron scattering. Furthermore, the effective ionization fraction can also be estimated by 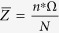, (where *N* being the number of ions in the cell). At the temperature of 60 kK and density of 5 g/cm^3^, a point around the metallization boundary, the effective ionization fraction is estimated to be about 1 represents a 50 percentage ionization. As the densities are larger than 14 g/cm^3^, all the estimated effective ionization fractions tend to be 2 which suggests an adequately second order ionization of helium. The results of Winisdoerffer *et al*.’s prediction by chemical free energy model^41^ suggested that pressure ionization occurred directly from atomic helium He to fully ionized helium He^2+^ at around 10 g/cm^3^, which is close to our estimation.

**Figure 4:**
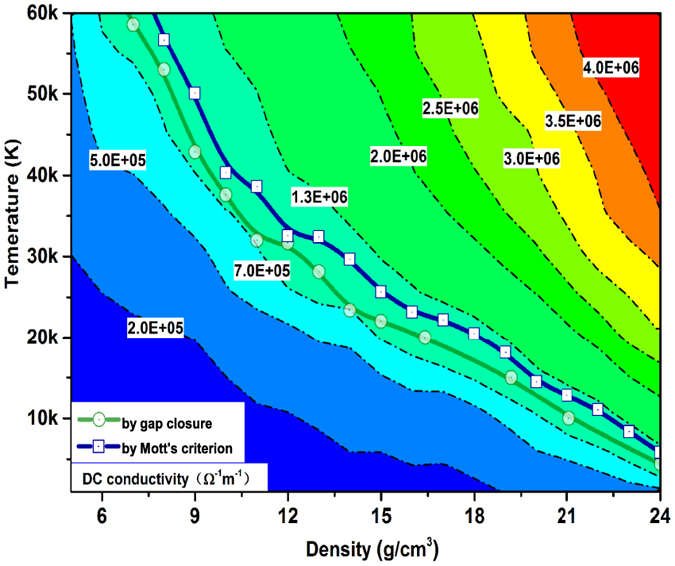
DC conductivity of helium in the *ρ* − *T* profile with a color scale together with its contours presented with dash dot curves and metallization boundary determined by gap closures (circles) and Mott’s criterion(squares) with solid line.

In the Mott picture of metallization process, the minimum conductivity critical value can be given by 
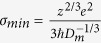
 42, where *D*_*m*_ is the atom density at metalized point, and *z* is the number of conduction electrons per atom. Follow the estimation of effective ionization fraction, taking *z* as 1, we can determine another new metallization boundary by Mott criterion, which is illustrated in Figs 2 and 4 together with the one determined by gap closure. It can be found that they are consistent with each other very well. Besides, in Fig. 4, the DC conductivity in wide density and temperature scope are illustrated in color scale and contours. Along the metallization boundaries, the DC conductivities are found to be 7.0 × 10^5^~1.3 × 10^6^ Ω^−1^m^−1^. Experimentally, other typical low Z molecular fluid (H, N, O) become poor metals with a conductivity of 2 × 10^5^ Ω^−1^m^−1^ 6,7,43,44,45. The fluid helium under such *ρ* − *T* conditions along the determined boundary can be considered as good conductor.

### Optical reflectivity

**Figure 5:**
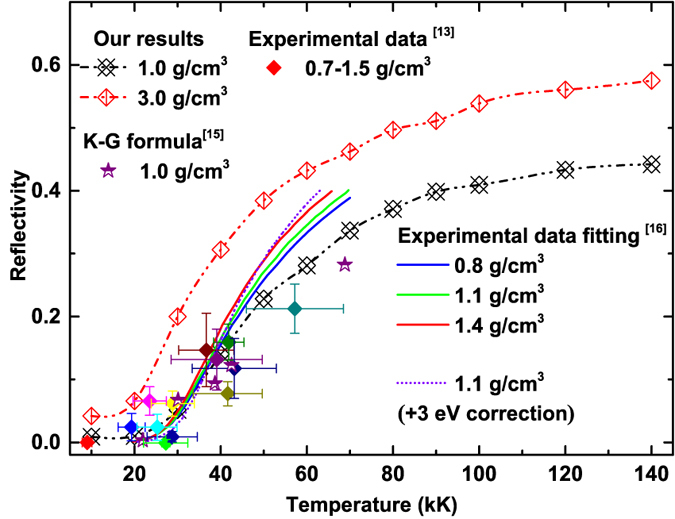
The calculated temperature dependence of the reflectivity of fluid helium at 1 and 3 g/cm^3^ together with other theoretical and experimental data for comparison.

Experimentally, the optical reflectivity probed in dynamic compression is also considered to be an important signature of metallization of impacted samples, which can be derived theoretically from the obtained dynamic conductivity. In Fig. 5, we show the variation of optical reflectivity at 532 nm wavelength of fluid helium with temperature at 1 and 3 g/cm^3^ together with other theoretical and experimental results for a comparison^13^,^15^,^16^. Under high temperature (more than 40 kK), the reflectivity given by Soubiran *et al*.^16^ are obviously higher than ours and experimental data^13^, especially the result with a 3 eV correction to the GGA gap energy as proposed by Kowalski *et al*.^15^. From this point of view, this also indicates that the underestimation of the band gaps using density functional theory is not significant at finite temperature.

**Figure 6:**
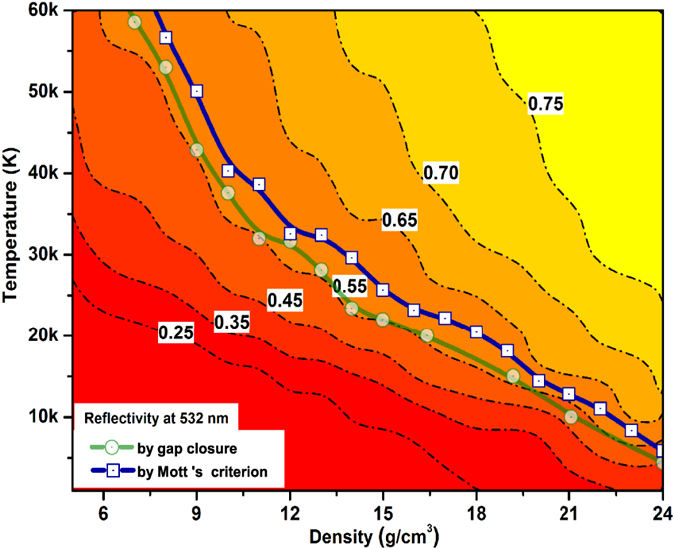
The calculated optical reflectivity at 532 nm in the *ρ* − *T* profile with a color scale together with its contours presented with dash dot curves and metallization boundary determined by gap closures (circles) and Mott’s criterion (squares) with solid line.

From Fig. 5, it can be found that the reflectivity exhibits a strong temperature dependence at start and tends to be saturation under high temperature, this corresponds to the process of increasing ionization and an insulator-to-metal transition (IMT). Such a behavior of steep reflectivity increase and saturation associates IMT in materials had been identified in earlier shock wave experiments on deuterium^44^, diamond^46^, polystyrene^47^ and etc. Recently, Knudson *et al*. had also showed a direct observation of an abrupt IMT in dense liquid deuterium from the evidence of a dramatic increase in reflectivity of the deuterium samples at the Sandia Z machine^48^. In Fig. 6, we plotted the reflectivity in the density and temperature plane with contours. The transformation of insulating molecular fluid to conducting fluid are often associated with the high optical reflectance. In this work, the reflectivity value of helium along the metallization boundary is found to be about 0.55, quite comparable to the measured reflectivity of conducting fluid produced by shock compression^44^,^46^,^47^,^48^, which can be severed as an useful theoretical reference to the optical diagnosing in future dynamic compression experiments on the metallization of warm dense helium.

### Conclusion

In conclusion, by extensive QLMD simulations as well as electronic structure properties calculations, we have proposed a *ρ* and *T* dependent formula for the helium gap value and built a reliable metallization boundary of fluid helium in a very wide range of temperature and density. The calculated electrical conductivity and optical reflectivity from liner response theory show good agreement with other theoretical and experimental results. Estimated effective ionization fractions by the Drude model suggest the second order ionization of helium as the density is larger than 14 g/cm^3^. Compare to previous experimental estimation, our results motivates new experiments to produce higher temperature or more dense helium to acquire metallic helium.

## Additional Information

**How to cite this article:** Zhang, W. *et al*. Revisiting metallization boundary of warm dense helium in a wide *ρ*-*T* regime from ab initio study. *Sci. Rep.* **7**, 41885; doi: 10.1038/srep41885 (2017).

**Publisher's note:** Springer Nature remains neutral with regard to jurisdictional claims in published maps and institutional affiliations.
