# Corrigendum: Revisiting metallization boundary of warm dense helium in a wide *ρ*-T regime from ab initio study

**DOI:** 10.1038/srep44968

**Published:** 2017-05-04

**Authors:** Wei Zhang, Zhiguo Li, Zhijian Fu, Jiayu Dai, Qifeng Chen, Lingcang Cai

Scientific Reports 7: Article number: 4188510.1038/srep41885; published online: 02 03 2017; updated: 05 04 2017

In the original version of this Article, Lingcang Cai was incorrectly listed as being affiliated with ‘School of Science, Southwest University of Science and Technology, Mianyang, 610064, Sichuan, P. R. China’. The correct affiliation is listed below:

Laboratory of Shock Wave and Detonation Physics, Institute of Fluid Physics, Chinese Academy of Engineering Physics, P.O. Box 919-102, Mianyang 621900, Sichuan, P. R. China.

This error has now been corrected in the HTML and PDF versions of the Article.

